# A Comparative Study of Eu^3+^-Doped Sillenites: Bi_12_SiO_20_ (BSO) and Bi_12_GeO_20_ (BGO)

**DOI:** 10.3390/ma16041621

**Published:** 2023-02-15

**Authors:** Marcin Kowalczyk, Marcin Kaczkan, Andrzej Majchrowski, Michał Malinowski

**Affiliations:** 1 Institute of Microelectronics and Optoelectronics, Warsaw University of Technology, Koszykowa 75, 00-662 Warsaw, Poland; 2 Institute of Applied Physics, Military University of Technology, Kaliskiego 2, 00-908 Warsaw, Poland

**Keywords:** Eu^3+^ luminescence, spectroscopy, sillenites, Bi_12_SiO_20_, Bi_12_GeO_20_

## Abstract

The spectroscopic properties of Eu^3+^-doped Bi_12_SiO_20_ (BSO) were investigated and compared with that of Eu^3+^-doped Bi_12_GeO_20_ (BGO). The emission properties and the absorption spectra have been measured at 10 K as well as at 300 K (room temperature). Luminescence was detected due to the direct excitation of the ^5^D_0_ level of Eu^3+^, as well as through the excitation of the ^5^D_1_ level. The Judd–Ofelt theoretical framework was used to compute the radiative lifetimes (τ) and the omega parameters (Ω_λ_). The electric dipole transition probabilities, asymmetry ratios (R), along with the branching ratios (β) were also determined based on the obtained experimental data. The strongest detected luminescence belongs to the ^5^D_0_ → ^7^F_0_ transition observed at 578 nm, similar to the BGO sillenite. Reasons for the major presence of the ^5^D_0_ → ^7^F_0_ emission, theoretically forbidden by the Judd–Ofelt Theory, were investigated and compared with that of the BGO sillenite. Obtained results showed that the strong ^5^D_0_ → ^7^F_0_ line is also present in Eu:BSO, indicating that this is a feature of the entire sillenite family and not just Eu:BGO.

## 1. Introduction

The Bi_12_SiO_20_ (BSO) and Bi_12_GeO_20_ (BGO) crystals are both members of the sillenite family of materials, which possesses a cubic cell symmetry and belong to the space group I23 (number 197). The lattice constant equals 10.1455 Å for BGO and 10.104 Å for BSO, with cell volumes equal to 1044.288244 Å^3^ and 1031.5 Å^3^ for BGO and BSO, respectively, as reported in the literature [1,2]. Both BGO and BSO crystals in their undoped form are well-researched materials, (the first experiments were performed over 55 years ago on BGO in 1967 by Abrams S.C. et al. [3]) with many practical applications [1,2,3,4,5,6,7,8,9,10,11,12,13,14], such as Pockels cells and sensors [1,3], phase-conjugated spatial-time light modulators [6], or holographic memory storage [13,14,15,16]. Over the last few years, doping of sillenites with transition-metal ions (e.g., Fe, Co, Cr or Cu) has been researched quite intensively with the goal of increasing the usefulness of BSO and BGO for holographic storage applications [17,18,19,20,21,22,23,24]. In 2020, BGO crystals doped with Eu^3+^ were successfully grown, which exhibited a strong luminescence in the yellow–red range of spectrum and could potentially be used as a laser material [25].

**Figure 1:**
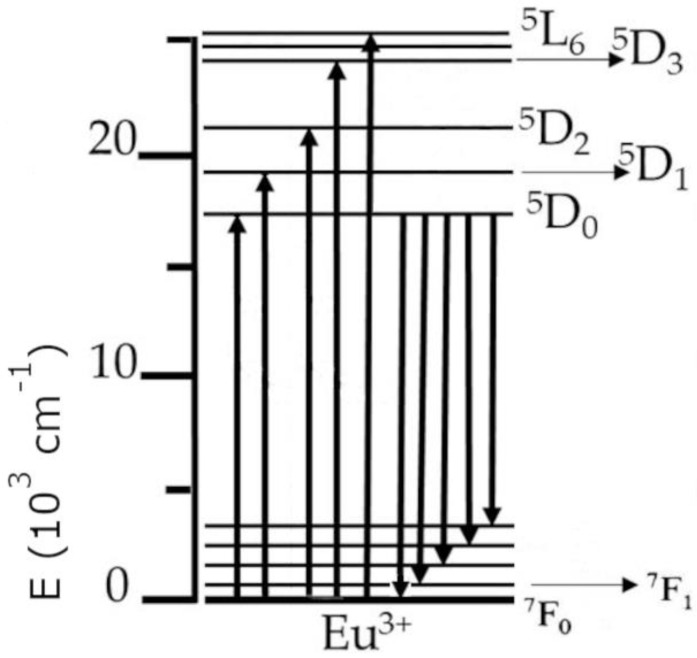
The partial energy diagram of the trivalent europium ion.

In principle, the luminescence process happens as follows: When the excitation radiation is being absorbed by a material, an electron moves from the ground state to a higher lying (upper) state. As the electron then returns from the upper state to the ground state, it emits a photon, which yields in turn observable luminescence in the host material. The trivalent europium ion (Eu^3+^) can be used in the investigations of various properties of dielectric materials (as a spectroscopic probe) due to the fact that the ^5^D_0_ energy level (which is a higher lying state, from which luminescence primarily occurs) and the ^7^F_0_ ground state of trivalent europium are of a nondegenerate nature, which results in a single observable line for transitions between those levels in the spectrum. This can be seen in the partial energy diagram for trivalent europium in Figure 1, where upward arrows signify excitation energy, while downward arrows symbolize emission. The other unique feature of Eu^3+^ is that the intensity of the ^5^D_0_ → ^7^F_1_ transition is independent of the environment (due to being of a magnetic dipole origin), which means it can be used as a reference to other transitions during analysis of the emission spectra [26].

During the study of Bi_12_GeO_20_ samples doped with europium, a very narrow and strong ^7^F_0_ emission line from the ^5^D_0_ level was observed [25]. As typically this line is very weak in most materials, additional study was warranted to see if this property is unique only to the Eu^3+^:Bi_12_GeO_20_ (Eu:BGO) or if it is a feature of the entire sillenite family when doped with europium. In light of the very interesting results of the Eu:BGO investigation [25], we have applied the Judd–Ofelt theory [26,27] in order to analyze the Eu^3+^:Bi_12_SiO_20_ (Eu:BSO) crystal system excited states dynamics and photoluminescence, in order to compare the Eu^3+^-doped BGO and Eu^3+^-doped BSO members of the sillenite family, to better understand the unique emission characteristics of Eu^3+^ in the I23 crystal system, and to further evaluate the potential benefits of doping sillenites with rare earths.

As only in the case of trivalent europium, the doubly reduced matrix elements are not equal to zero for only one parameter (U^2^) per a given transition, which allows the calculation of the Judd–Ofelt intensity parameters (Ω_2,4,6_) in a relatively simple manner, by using only the emission spectrum, which is impossible for any of the other rare-earth ions. The Judd–Ofelt parameters permit the calculations of other crucially important factors, such as the probabilities of the emission transitions, which is significant, for example, during the evaluation of a material as a possible candidate for lasing [26,27].

## 2. Materials and Methods

### 2.1. Crystal Growth

BSO melts congruently at 895 °C, therefore, it can be directly grown from melts of stoichiometric composition (Bi_2_O_3_:SiO_2_ in molar ratio equal to 6:1) [28]. In our investigations, we carried out single crystal growth of BSO:Eu by means of the Kyropoulos method [29]. Good-quality pure BSO single crystals grown in the past [28] by means of the Chochralski technique [28,30] were used as a raw material. The BSO single crystals grown with the use of 99.99% Bi_2_O_3_ and SiO_2_ were transparent and did not have any imperfections that could be seen during visual inspection. They were crushed and molten with a Eu_2_O_3_ powder of 99.99% purity. 1 at. % of Eu substituting Bi ions was used. The Kyropoulos technique, due to properly established temperature gradients in a two-zone resistance furnace controlled with 2704 Eurotherm regulators/programmers, allowed the growth of seeded bulk single crystals in the volume of the melt. No pulling was used, the rotation rate was equal to 6 rpm, and the furnace temperature was lowered at a rate of 0.02 K/h. The crystallization was carried out with the use of a BSO seed oriented in a [110] crystallographic direction. After reaching approximately 30 mm in the cross-section, the as-grown BSO:Eu single crystal was pulled out of the melt and cooled to room temperature at the rate of 6 K/h.

**Figure 2:**
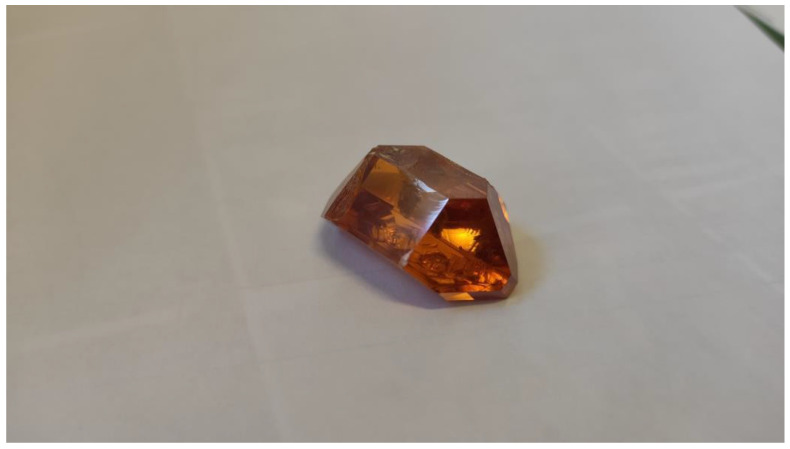
Bottom and side faces of the [110] BSO:Eu single crystal, as grown.

**Figure A1:**
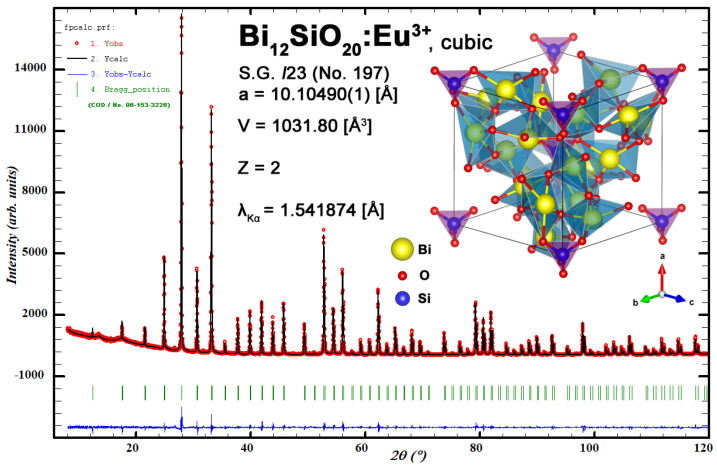
Experimental XRD powder diffraction pattern along with Rietveld refinement plot and refined structure of Bi_12_SiO_20_:Eu^3+^ unit cell.

Relatively low temperature gradients allowed the growth of BSO:Eu single crystals confined with crystallographic faces, as shown in Figure 2, where the bottom and side faces of as-grown [110] BSO:Eu single crystal are shown. Two-fold symmetry can be clearly seen. The crystal was transparent except for a narrow core in the central part of the as-grown crystal near the bottom face. The formation of the core was caused by constitutional supercooling due to a lowering of the temperature gradient on the crystal bottom ((110) surface) propagating into the volume of melt. The experimental XRD powder diffraction pattern along with the Rietveld refinement plot and refined structure of the Bi_12_SiO_20_:Eu^3+^ unit cell is shown for reference in Figure A1, and matches undoped sillenite reference data (per Crystallography Open Database entry ID 1533225).

### 2.2. Samples Characterization

Emission measurements were carried out using a spectrophotometer manufactured by Photon Technology International (Photon Technology International, Edison NJ, USA). Emission spectra were measured with a double set monochromator (model: SP-2500i Manufacturer: Teledyne Princeton Instruments, Acton, MA, USA), which was followed by a photomultiplier tube (PMT) and a photon counting system (model: SR-400 Manufacturer: Stanford Research Systems, Sunnyvale, CA, USA). The samples were also excited by a pulsed tunable optical parametric oscillator (operating at a 10 ns pulse width, with a repetition rate of 10 Hz. Made by: Continuum (now part of Amplitude Laser company, Amplitude Laser Inc., Milpitas, CA, USA)) which was pumped by a frequency-tripled Nd: YAG pulse laser (model: Continuum Surelite II, also manufactured by Amplitude Laser Inc., Milpitas, CA, USA). For room temperature studies, the samples were excited with a optically pumped semiconductor laser (OPSL) (Verdi G8 made by Coherent Inc., Santa Clara, CA, USA ) operating at 532 nm in a CW mode. The absorption spectrum was measured at room temperature (300 K) using a spectrophotometer (make and model: Lambda 950 manufactured by Perkin–Elmer, Llantrisant, UK). Fluorescence dynamics profiles were recorded with a multi-channel analyzer (model: SR-430 Manufacturer: Stanford Research Systems, Sunnyvale, CA, USA ) controlled with a PC. The sample was cooled using a closed-cycle He optical cryostat system (Displex ARS CSW-202, made by Advanced Research Systems, Macungie, PA, USA) which permitted the temperature to be adjusted from 300 K to 10 K.

## 3. Results and Discussion

**Figure 3:**
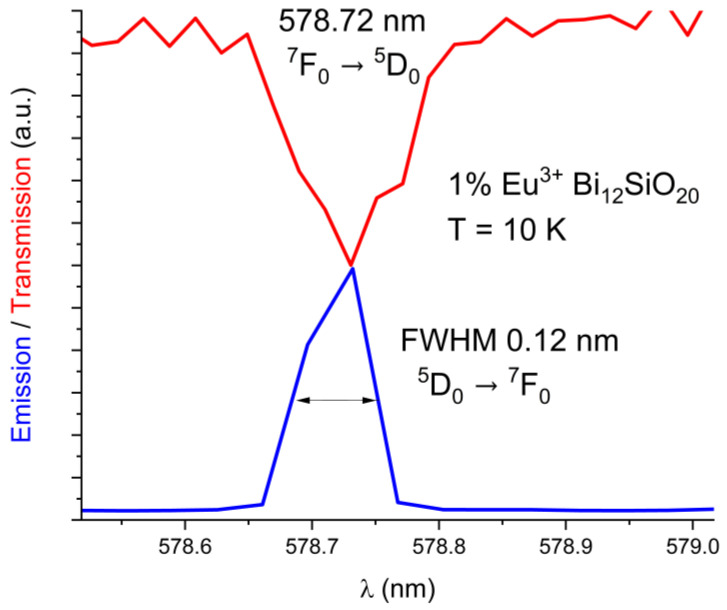
Low-temperature (10 K) absorption line ^7^F_0_ → ^5^D_0_ overlaid with the ^5^D_0_ → ^7^F_0_ emission line in BSO.

The emission and absorption spectra were measured at 10 K and 300 K with the aid of the cryostat equipment. The BSO crystal, similar to the BGO crystal, exhibits strong absorption in the short-wavelength region of the visible spectrum. The exact location of the ^5^D_0_ ← ^7^F_0_ level of Eu^3+^ for BSO has been determined from the 10 K absorption spectrum to be 17279 cm^−1^ (578.72 nm), while for BGO it was previously determined to be 17277 cm^−1^ (578.8 nm) [25]. Only one maximum for the ^5^D_0_ ← ^7^F_0_ line has been detected in both Eu^3+^-doped BSO and BGO. The line spectral widths are comparable in both BGO and BSO. The comparison between emission and absorption lines in BSO is shown below in Figure 3. (absorption line in red, emission line in blue). The FWHM is equal to 0.12 nm in BSO.

**Figure 4:**
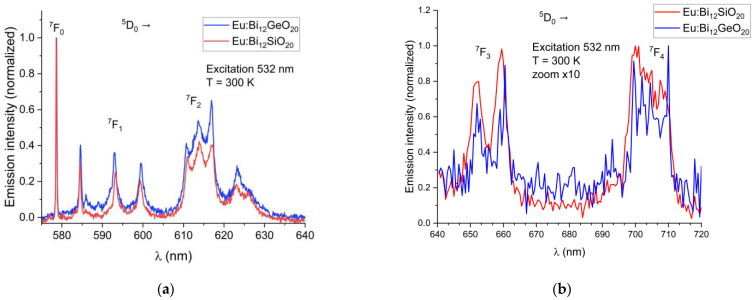
(**a**) Partial emission spectra of Eu^3+^ showing the ^5^D_0_ → ^7^F_0,1,2_ transitions in BSO and BGO at T = 300 K; (**b**) Partial emission spectra of Eu^3+^ showing the ^5^D_0_ → ^7^F_3_ and ^5^D_0_ → ^7^F_4_ transitions in BSO and BGO at T = 300 K (scaled for better visibility).

Emission spectra of the BSO sample were recorded at 10 K and at 300 K. To compare the spectra of BSO and of BGO correctly, all the emission spectra were normalized with regards to their maximum emission intensity, scaled to the range from 0 to 1. The emission spectra of Eu^3+^ from the ^5^D_0_ level for BSO (this work) and BGO [25] recorded at room temperature are shown in Figure 4a,b below, where all transitions except a very rarely observed ^5^D_0_ → ^7^F_6_ and ^5^D_0_ → ^7^F_5_ transition are presented. During the investigations of the Eu^3+^-doped BGO samples [25], it was discovered that, while shorter-wavelength visible light is strongly absorbed by the host matrix, thus preventing efficient direct excitation of europium, there exist two alternatives: indirect through UV excitation of Bi^3+^ at 365 nm and thus activation of Eu^3+^ through energy transfer; or through the direct Eu^3+^ excitation at a wavelength of 532 nm (^5^D_0_ excitation through the ^5^D_1_ level from the ^7^F_1_ state), which is very efficient at room temperature because the ^7^F_1_ state can be thermally populated at room temperature due to its very close proximity to the ^7^F_0_ ground state [31,32]. The same transitions are visible when exciting Eu^3+^ directly or via energy transfer from Bi^3+^. Direct europium excitation via 532 nm yields an emission of a higher intensity relative to the energy transfer method.

**Figure 5:**
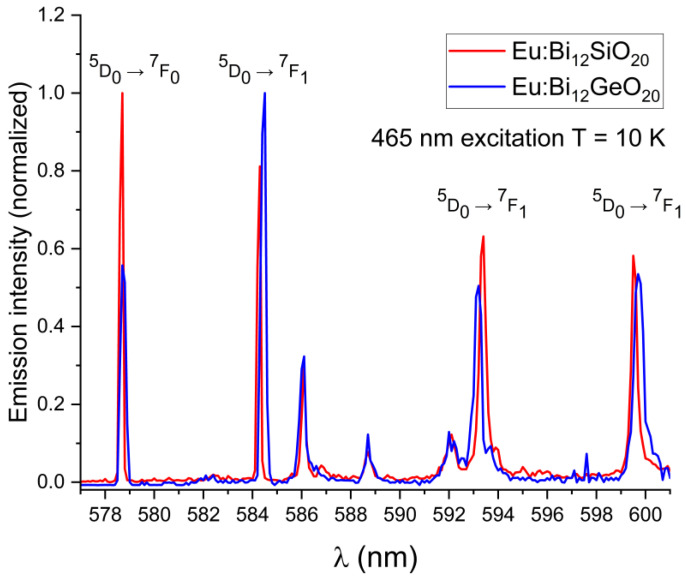
Low-temperature emission spectra (at T = 10 K) showing the wavelength shift for the primary ^5^D_0_ → ^7^F_0,1_ transitions of Eu^3+^ in BSO with regards to BGO; weak lines from the ^5^D_1_ level in the range 586 nm to 592 nm are also visible.

In both BSO and BGO, the ^5^D_0_ → ^7^F_0_ transition is very narrow and is the strongest of all observed transitions under the 532 nm excitation at room temperature, with the ^5^D_0_ → ^7^F_1_ and ^5^D_0_ → ^7^F_2_ having a higher intensity in BGO than in BSO (as can be seen in Figure 4a). The ^7^F_1_ levels are triply split in both BGO and BSO at 300 K and at 10 K, which confirms the same crystal symmetry in both specimens, as expected, with the ^7^F_1_ and ^7^F_2_ peaks shifted by about 0.1 nm in BSO with respect to BGO when measured at room temperature. Since the ^7^F_1_ level becomes thermally depopulated at very low temperatures, this prevents the use of the 532 nm excitation wavelength for the low-temperature studies at 10 K; less efficient excitation via the wavelength of 465 nm is therefore used instead, since it was experimentally proven that both BGO [25] and BSO exhibit luminescence under that excitation wavelength at 10 K, albeit with a lesser intensity than when excited at room temperature. Weak ^5^D_1_ emissions were observed in the range of 586 nm to 592 nm because of the ^5^D_1_ level excitation. Low-temperature (10 K) emission spectra showing the major ^5^D_0_ → ^7^F_0,1,2_ transitions in BSO and BGO, along with weak emissions from the ^5^D_1_ level are shown in Figure 5. The ^7^F_0_ emission line is not split in both BSO and BGO, indicating that the europium ion occupies only a single site in both cases.

**Figure 6:**
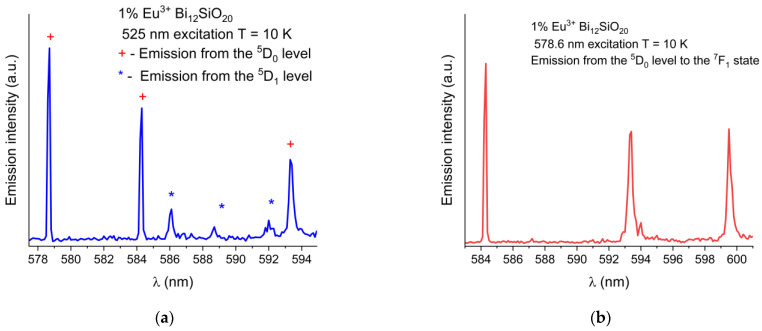
(**a**) Partial low-temperature (10 K) emission spectrum of Eu^3+^ in BSO showing combined ^5^D_0_ and weak ^5^D_1_ emission lines under 525 nm excitation. (**b**) Emission from the ^5^D_0_ level to the ^7^F_1_ state, under resonant ^5^D_0_ level excitation at 578 nm, showing no lines present in 586 nm to 592 nm range.

The wavelength shift of the Eu^3+^ emission peaks in BSO with respect to BGO is more pronounced at low temperatures (the ^7^F_1_ peak at 584.3 nm is blue-shifted by 0.2 nm, the ^7^F_1_ peak at 593.4 nm is red-shifted by 0.2 nm, and the ^7^F_1_ peak at 599.5 nm is blue-shifted by 0.2 nm). While the ^5^D_0_ → ^7^F_0_ emission peaks were of equal intensity at room temperature, at low temperature under the 465 nm excitation, the peak remains strongest in BSO, while in BGO, the peak intensity decreases significantly (albeit with no noticeable shift in position with respect to the same peak in BSO when measured at low temperature), and the ^5^D_0_ → ^7^F_1_ line is dominant instead. This indicates that nonradiative and thermal processes have a significant impact on the emission characteristics of trivalent europium in both sillenites. In order to verify the origin of the weak emission lines seen in Figure 5, partial emission spectrum were taken under 525 nm excitation to exclude any possible emissions from higher states, permitting only the emissions from the ^5^D_0,1_ levels to occur. Next, the excitation wavelength was changed to 578 nm to permit only emissions from the ^5^D_0_ level to occur. The partial spectrum with the ^5^D_0,1_ lines shown in detail is presented in Figure 6a, while in Figure 6b, only the triple ^7^F_1_ lines resulting from direct ^5^D_0_ excitation are shown. While the weak lines in the range 586 nm to 592 nm are present in the emission spectrum taken under the 525 nm excitation, they are absent from the spectrum where only the ^5^D_0_ was excited directly, thus confirming that the weak lines in question are present due to the emission from the ^5^D_1_ level.

**Figure 7:**
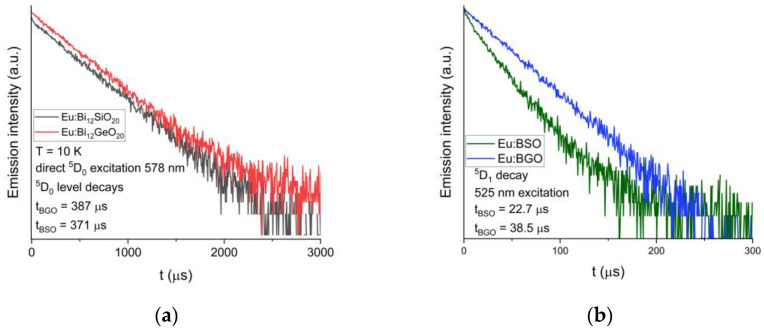
(**a**) Comparison of the decay curves for the ^5^D_0_ level of trivalent europium in BSO and BGO; recorded at T = 10 K. (**b**) Comparison of the decay curves for the ^5^D_1_ level of trivalent europium in BSO and BGO; recorded at T = 10 K.

The luminescent decay of the ^5^D_0_ level was also measured at T = 10 K. The decay time is equal to 371 μs for BSO, while for BGO it is equal to 387 μs [25], as can be seen in Figure 7a below. Additionally, the calculated theoretical radiative luminescence decay times both for BGO and BSO are significantly longer than experimentally measured, which can be attributed to the presence of a very intense ^5^D_0_ → ^7^F_0_ line, the contribution of which is not accounted for by the classical Judd–Ofelt theory [27,33,34], as discussed later in this article.

Luminescent decays of the ^5^D_1_ level under the 525 nm excitation were also compared between BGO and BSO, and similarly to the ^5^D_0_ level, the ^5^D_1_ level lifetime measured in BSO (equal to 23 μs) is shorter than that of BGO (equal to 39 μs [25]).

**Figure 8:**
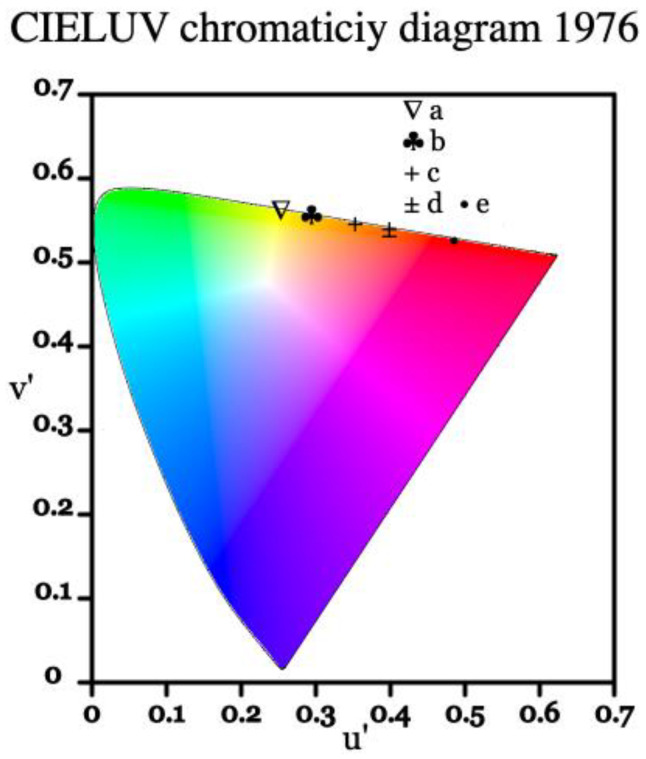
CIE 1976 representation of significant emission lines from the ^5^D_0_ level in Eu:BSO, where points from *a* to *e* represent significant peaks. Point *a* represents the ^5^D_0_ → ^7^F_0_ transition, points *b* to *d* represent the ^5^D_0_ → ^7^F_1_ transition, and point *e* represents the ^5^D_0_ → ^7^F_2_ transition.

The luminescence of a material can also be presented as a set of coordinates within the CIE color coordinate system, which is commonly used as a framework referencing how a human eye perceives color [35]. Below in Figure 8, the emission profile of europium-doped BSO is represented on a CIE 1976 chromaticity diagram. Similar to the Eu:BGO samples, due to the high intensity of the ^5^D_0_ → ^7^F_0_ transition and moderately intense ^5^D_0_ → ^7^F_1_ transition, the spectrum is of yellow-orange tint, as opposed to the typical deep-red found in Eu^3+^-doped materials where ^5^D_0_ → ^7^F_2_ transition dominates.

The Judd–Ofelt theory [27,33,34] is a recognized method for comparison and analysis of the spectroscopic properties of trivalent rare-earth ions (also known as lanthanides) in dielectric hosts. For most of those elements, calculations are based upon the tabulated reduced matrix elements (U^2^) and measurements of integrated absorption cross sections. By using the convention and symbols based upon the formulas derived by K. Binnemans in [26], the dipole strength (in units of Debye^2^), based upon the integrated peak areas of the absorption spectra, can be expressed as:

(1) $$D_{exp}=\frac{1}{108.9*C*d*X_{A}\left(T\right)}*\int^{\;}\frac{A(\tilde{\nu )}}{\tilde{\nu }}d\tilde{\nu }$$

where $\tilde{\nu }$ denotes the mean wavenumber of the transition (in cm^−1^), *C* is the mol dopant concentration, *d* symbolizes the optical path length, and $X_{A}\left(T\right)$ is the fractional thermal population at temperature *T* of level *A* (from which the absorption process starts). Furthermore, the calculated dipole strength can be obtained by using the below formula:

(2) $$D_{calc}=\frac{10^{36}}{2J+1}*\chi_{ED}*e^{2}*\underset{\lambda =2,4,6}{\sum }\Omega_{\lambda }|<J||U^{\left(\lambda \right)}||{J^{\prime }>|}^{2}$$

where 2*J* + 1 denotes the degeneracy of the ground state, *e*^2^ is the value of elementary charge, and 10^36^ is the factor to convert from the *D*^2^ into esu × cm. $|<J||U^{\left(\lambda \right)}||{J^{\prime }>|}^{2}$ are the squared reduced matrix elements (which were tabulated and made widely available through the works of Carnall et al. [36,37]). The term $\chi_{ED}=\frac{{\left(n^{2}+2\right)}^{2}}{9n}$ accounts for the correction of the effect in the dielectric medium, where n denotes its refractive index. As the Judd–Ofelt calculations are often completed using the oscillator strength parameter, oscillator strength *f* and the dipole strength *D* can be converted by using the following formula:

(3) $$D=\frac{2.124*10^{6}f}{\tilde{\nu }}$$

It is significant to remark at this point that the trivalent europium ion has most of the reduced matrix elements (U^2^) equal to 0. The only values greater than zero are for: ║U^2^║^2^ element, which corresponds to the ^5^D_0_ →^7^F_2_ transition; as well as for ║U^4^║^2^ and║U^6^║^2^ elements, which correspond to the ^5^D_0_ → ^7^F_4_, and ^5^D_0_ → ^7^F_6_ transitions, respectively. Therefore, it is feasible to calculate the values of the Ω_λ_ parameters for europium-doped materials based upon the integrated areas of the emission spectrum. As the ^5^D_0_ → ^7^F_1_ transition is independent of the host environment (due to its magnetic-dipole character), it can therefore be treated as a reference for other transitions originating from the ^5^D_0_ excited state. Based upon the formulas published by Binnemans K. [26], the reference value for the ^5^D_0_ → ^7^F_1_ transition (*A_ref_*) may be calculated as follows:

(4) $$\frac{1}{\tau_{rad}}=A_{MD,0}n^{3}*\left(\frac{I_{tot}}{I_{MD}}\right)$$

where *A*_*MD*,0_ denotes the spontaneous emission probability for the ^5^D_0_ → ^7^F_1_ magnetic dipole transition in vacuo (equal to 14.65 s^−1^ [26]), and *n* denotes the refractive index of the host material. It is therefore possible to derive the Judd–Ofelt omega parameters (Ω_λ_) from the ratio of the integrated intensity of the ^5^D_0_ → ^7^F_J_ (where *J* = 2, 4 or 6) transitions (denoted as $\int^{\;}I_{\lambda }\left(\tilde{\nu }\right)d\tilde{\nu }$ in the below equation) to the ratio of the integrated intensities of the ^5^D_0_ → ^7^F_1_ transition (marked as $\int^{\;}I_{1}\left(\tilde{\nu }\right)d\tilde{\nu }$ in the below equation) by using the formula [26]:

(5) $$\Omega_{\lambda }=\frac{D_{MD}{\tilde{\nu }}_{1}^{3}}{e^{2}{\tilde{\nu }}_{\lambda }^{3}{\left|<\Psi J||U^{\left(\lambda \right)}||\Psi J^{\prime }>\right|}^{2}}*\frac{9n^{3}}{n{\left(n^{2}+2\right)}^{2}}*\frac{\;\int^{\;}I_{\lambda }\left(\tilde{\nu }\right)d\tilde{\nu }\;}{\int^{\;}I_{1}\left(\tilde{\nu }\right)d\tilde{\nu }}$$

where the ${\tilde{\nu }}_{1}$ symbolizes the averaged wavenumber of the ^5^D_0_ → ^7^F_1_ transition and ${\tilde{\nu }}_{\lambda }$ symbolizes the average wavenumber of the ^5^D_0_ → ^7^F_2,4,6_ transitions. The averaged wavenumber can be calculated as follows:

(6) $${\tilde{\nu }}_{\lambda }=\frac{\int^{\;}\tilde{\nu }I\left(\tilde{\nu }\right)d\tilde{\nu }}{\int^{\;}I\left(\tilde{\nu }\right)d\tilde{\nu }}$$

As the ^5^D_0_ → ^7^F_6_ transition is observed very rarely, it is often just possible to derive the $\Omega_{2}$ and $\Omega_{4}$ Judd–Ofelt parameters but not the $\Omega_{6}$. Given that the ^5^D_0_ → ^7^F_1_ transition has a magnetic-dipole character, its dipole strength can therefore be expressed as D_MD_ = 9.6 * 10^−6^ Debye^2^, and upon further assumption that the D_MD_ is equal to 0 for other transitions, then for transitions ^7^F_2,4,6_ from the ^5^D_0_ level, the D_ED_ factor is calculated as follows:

(7) $$D_{ED}=e^{2}\underset{\lambda =2,4,6}{\sum }\Omega_{\lambda }{\left|<\Psi J||U^{\left(\lambda \right)}||\Psi J^{\prime }>\right|}^{2}$$

Additionally, for the ^5^D_0_ → ^7^F_0,3,5_ transitions, both D_MD_ and D_ED_ values are assumed to be 0 [26]. Considering all of the above, it is therefore possible to derive the radiation transition probabilities for all of the excited states by using the calculated $\Omega_{\lambda }$ parameters of the Judd–Ofelt theory by using the following equation [26]:

(8) $$A\left(\Psi J,\Psi^{\prime }J^{\prime }\right)=\frac{64\pi^{4}{\tilde{\nu }}^{3}}{3h\left(2J+1\right)}*\left[\frac{n{\left(n^{2}+2\right)}^{2}}{9}*D_{ED}+n^{3}D_{MD}\right]$$

where the average wavenumber of the transition (in units of cm^−1^) is denoted by $\tilde{\nu }$, 2*J* + 1 denotes the degeneracy of the initial state, and *h* symbolizes the Planck constant. Therefore, the radiative branching ratios β_R_($\Psi J,\Psi^{\prime }J^{\prime })$ from level *J* to *J*′ can now be derived by using the $A\left(\Psi J,\Psi^{\prime }J^{\prime }\right)\;$ values from the formula above [26] since:

(9) $$\beta_{R}\left(\Psi J,\Psi^{\prime }J^{\prime }\right)=\frac{A\left(\Psi J,\Psi^{\prime }J^{\prime }\right)}{\sum_{\Psi^{\prime }J^{\prime }}A\left(\Psi J,\Psi^{\prime }J^{\prime }\right)}$$

Through the application of the standard least-squares method, the root mean square (RMS) deviation can be defined as shown below:

(10) $$RMS=\sqrt{\frac{\sum_{i}{\left(D_{exp}^{i}-D_{calc}^{i}\right)}^{2}}{N-3}}$$

where *N* is the number of transitions used in the fitting procedure and 3 is the number of the parameters being fitted ($\Omega_{2},\Omega_{4},\Omega_{6})$. It is therefore possible at this point to fit iteratively the $\Omega_{\lambda }$ parameters. Additionally worth mentioning is the asymmetry parameter R, which is defined as the ratio between the integral intensities of the ^5^D_0_ → ^7^F_2_ and ^5^D_0_ → ^7^F_1_ transition bands and can be calculated from the emission spectrum recorded at room temperature (300 K). It can be formally written as *R* = *I*(^5^*D*_0_ → ^7^*F*_2_)/*I*(^5^*D*_0_ → ^7^*F*_1_) and can indicate the trivalent europium ion symmetry. Specifically, the further from a centrosymmetric geometry the luminescent center is located, the larger is the expected the value of the R parameter [26,38].

The R parameter equals 2.347 for the 1% Eu^3+^-doped BGO sample [25], and 2.619 for BSO, also doped 1% Eu^3+^. The slight differences in the R values between similarly doped sillenites can be accounted for by the fact that the ionic radius of Si is less than that of Ge, which affects the length of the atomic bonds in the primary structure of the sillenite, therefore influencing the internal stability [21]. To a lesser degree, there may be slight variations in the exact amount of europium dopant in each sample.

The Judd–Ofelt parameters and branching and asymmetric ratios were calculated for BSO based upon the recorded emission spectra. The parameters for BGO are sourced from [25]. The spontaneous emission probabilities, radiative lifetimes, and fluorescence branching ratios of BSO and BGO using the derived parameters are shown for comparison in Table 1.

Obtained theoretical radiative lifetimes are both, in the case of BSO and BGO, significantly longer than experimentally measured values, which indicates that not all of the processes that are occurring are being taken effectively into account by the classical Judd–Ofelt theory [27] (including thermal and other complex processes such as spin–orbit interaction or J–J mixing [26,39,40], which can have a significant impact on the emission characteristics of Eu^3+^ [26,41,42]). The comparison of the Judd–Ofelt parameters between europium-doped BSO and other trivalent europium-doped hosts is shown in Table 2 below. As mentioned above, the Judd–Ofelt parameter Ω_2_ can generally be used to represent the strength of the covalency and the site symmetry of europium [26,38,43]. The value of Ω_2_ in the BSO sample is larger than that of europium-doped hosts: Bi_12_GeO_20_,YAl_3_(BO_3_)_4_, Ba_2_GdV_3_O_11_, LaF_3_ and YAlO_3_. This trend further validates the stipulation that the values of, and relations between, the derived Judd–Ofelt omega parameters are strongly host-dependent, and thus can be used as a general indicator of the host symmetry, as stated in [25].

Additionally, based upon the results obtained from the emission spectra of trivalent europium in BSO, the energy level positions for the primary emissions from the ^5^D_0_ level were determined and are presented in Table 3 below.

The obtained results show a lot of similarities among Eu^3+^-doped BSO and Eu^3+^-doped BGO materials with respect to the emission spectrum, including the presence of a very strong ^5^D_0_ → ^7^F_0_ emission line. The difference between calculated Judd–Ofelt parameters for the materials under investigation is quite small—less than 1 for both Ω_2_ and Ω_4_ parameters. The observable luminescent decay times are also similar—differing by less than 20 μs. The R_JJ_ parameter, indicative of the degree of J–J mixing [39,40] which can be defined formally as *R_JJ_* = *I*(^5^*D*_0_ → ^7^*F*_0_)/*I*(^5^*D*_0_ → ^7^*F*_1_), is in the case of BSO, equal to 0.1052, whereas in case of BGO, it is 0.2962 [25]. Therefore, in light of the obtained results, it is reasonable to assume that the stipulations with regard to J–J mixing, spin–orbit interaction, [26,39,40,51] the Wybourne–Downer mechanism [52], and the breakdown of the closure approximation in the Judd–Ofelt theory [53] stipulated in the case of the ^5^D_0_ → ^7^F_0_ emission line in BGO [25] also hold true in case of europium-doped BSO. It would be an interesting future study to check whether other sillenite family members such as Bi_12_TiO_20_ (BTO) would show similar results. It would also be scientifically beneficial to investigate deeper the nonradiative and thermal-related interactions and their influence on the Eu^3+^ ion in the scope of the family of sillenites.

## 4. Conclusions

The spectroscopic properties of trivalent europium-doped Bi_12_SiO_20_ (BSO) sillenite bulk crystals were investigated. The emission properties as well as the absorption spectra have been measured at 300 K and at 10 K. Luminescence from the ^5^D_0_ level was observed both at room temperature (300 K) as well as at 10 K. The Judd–Ofelt omega parameters as well as the radiative lifetimes were successfully derived based upon the Judd–Ofelt theory. [27,33,34] Electric dipole transition probabilities and branching ratios were also determined based upon obtained experimental measurements. Similarities and differences between europium-doped BGO and BSO were discussed and potential further areas for scientific investigation have been outlined.

## Figures and Tables

**Table 1:** The spontaneous emission probabilities (A), radiative lifetimes (τ), fluorescence branching ratios (β), and asymmetry ratios (R) of BSO and BGO calculated using the obtained Judd–Ofelt parameters (Ω).

Transition	λ (nm) BSO	λ (nm) BGO	A_calc_ (s^−1^) BSO	A_calc_ (s^−1^) BGO	β_calc_ BSO	β_calc_ BGO
^5^D_0_ → ^7^F_0_	578	578	0	0	0	0
^5^D_0_ → ^7^F_1_	583	584	242.038	249.258	0.2187	0.2557
^5^D_0_ → ^7^F_2_	614	614	763.465	652.459	0.6899	0.6694
^5^D_0_ → ^7^F_3_	660	660	0	0	0	0
^5^D_0_ → ^7^F_4_	699	705	100.994	72.995	0.0912	0.0748
^5^D_0_ → ^7^F_5_	-					
^5^D_0_ → ^7^F_6_	-					
Σ			1106.497	974.672	1	1
$\Omega_{2}\left(\mathrm{BSO}\right)=$3.752 * 10^−20^ cm^2^			$\Omega_{2}\left(\mathrm{BGO}\right)=$3.122 * 10^−20^ cm^2^			
$\Omega_{4}\left(\mathrm{BSO}\right)=$1.095 * 10^−20^ cm^2^			$\Omega_{4}\left(\mathrm{BGO}\right)=$0.774 * 10^−20^ cm^2^			
τ_calc_ (BSO) = 903.753 μs			τ_calc_ (BGO) = 1025.9 μs			
R (BSO) = 2.619			R (BGO) = 2.347			

**Table 2:** The comparison of the Judd–Ofelt parameters between different europium-doped hosts.

Host	Ω_2_ (10^−20^ cm^2^)	Ω_4_ (10^−20^ cm^2^)	Ω_6_ (10^−20^ cm^2^)	Reference
Bi_12_SiO_20_	3.75	1.09	-	This work
Bi_12_GeO_20_	3.12	0.77	-	[25]
Bi_4_Ge_3_O_12_	4.39	2.70	0.64	[43]
KLu(WO_4_)_2_	20.76	5.23	7.96	[44]
KY(WO_4_)_2_	36.70	11.50	3.40	[45]
YAl_3_(BO_3_)_4_	2.26	5.11	0.78	[46]
Ba_2_GdV_3_O_11_	3.48	0.11	-	[47]
NaBi(WO_4_)_2_	3.95	0.10	-	[48]
LaF_3_	1.19	1.16	0.39	[49]
YAlO_3_	2.66	6.33	0.80	[49]
ZnO	9.59	8.11	0.25	[49]
Y_2_O_3_	9.86	2.23	0.32	[49]
Gd_2_O_3_	12.39	2.02	0.19	[50]

**Table 3:** Partial energy structure of Eu^3+^ determined from observed emission transitions from the ^5^D_0_ state for BSO and BGO hosts.

Level	Position in BSO (cm^−1^)	Position in BGO (cm^−1^)
^5^D_0_	17,280	17,280
^7^F_2_	1088	1083
	1046	1057
	996	1000
	978	976
	914	907
^7^F_1_	600	604
	428	422
	166	170
^7^F_0_ (ground state)	0	0

## Data Availability

Data can be provided upon a reasonable request.
